# DNA simulation benchmarks revealed with the accumulation of high-resolution structures

**DOI:** 10.1007/s12551-024-01198-2

**Published:** 2024-06-18

**Authors:** Wilma K. Olson, Robert T. Young, Luke Czapla

**Affiliations:** Rutgers, The State University of New Jersey, Piscataway, NJ USA

**Keywords:** DNA benchmarks, DNA deformability, DNA microenvironment, DNA sequence-dependent structure, Protein-DNA interactions

## Abstract

DNA carries more than the list of biochemical instructions that drive the basic functions of living systems. The sequence of base pairs includes a multitude of structural and energetic signals that determine the degree to which the long, threadlike molecule moves and how it responds to proteins and other molecules involved in its processing and packaging. The arrangements of successive base pairs in high-resolution protein-DNA crystal structures provide useful benchmarks for atomic-level simulations of double-helical DNA as well as information potentially useful in interpreting the properties of specific DNA sequences. The set of currently available structures has enough examples to characterize the conformational preferences of the DNA base-pair steps within the context of their immediate neighbors, i.e., in the context of tetramers, and reveals surprising effects of certain neighbors on local chain properties. The proteins in contact with DNA present various microenvironments that sense and/or induce the observed spatial forms. The cumulative buildup of amino-acid atoms in different protein-DNA complexes produces a binding cloud around the double helix with subtle sequence-dependent features. While the microenvironment presented by each protein to DNA is highly unique, the overall composition of amino-acid atoms within close range of DNA in a broad collection of structures is fairly uniform. The buildup of protein atoms of different types around the DNA provides new information for the improvement of nucleic acid force fields and fresh ideas for the exploration of the properties of DNA in solution.

## Introduction

The DNA double helix must deform in order to fit and function inside the tight confines of a cell. For example, the 2 m of DNA found in most human cells must be folded to fit in a nucleus of ~ 6 µm diameter (Alberts et al. 2022), and the base pairs must open to reveal the genetic instructions encoded along their hydrogen-bonded edges. The folding of overall structure entails large-scale bending of the double helix, and the separation of base pairs necessitates unwinding and displacement of the paired DNA strands.

The unique spatial arrangements of the double helix and the patterns of molecular association found in the ever-growing number of high-resolution DNA structures provide valuable insights into the molecular features that govern the processing and organization of the genetic material. Moreover, the sequence of bases dictates the degree to which the long, threadlike molecule deforms (Young et al. 2022) and how the duplex contacts and responds to the proteins and other molecules involved in its activity and packaging (Olson et al. 2022).

The vast majority of experimentally determined structures of DNA occur in the context of protein-DNA assemblies. The proteins impose widely different responses in the DNA, with the distortions introduced by a broad variety of proteins thought to reveal the natural rest states and conformational responses of the double helix (Olson et al. 1998). These features hint of pathways that may lead to extreme structural changes, such as atypical bending, twisting, and/or stretching and “melted” states with disrupted base pairing. The persistence of structural features within the same sequence context in numerous protein-DNA systems points to the underlying roles played by individual nucleotides in the organization and recognition of the double helix.

The spatial information in the observed structures also provides useful benchmarks for checking the state-of-the-art, atomic-level simulations increasingly employed in the treatment of DNA. The simulated structures are highly sensitive to the force fields that are applied to the individual nucleotides and are thus subject to the limitations of these treatments. DNA is difficult to deal with in that its double-helical structure is determined by both the local base-pair context and the long-range electrostatics of the sugar-phosphate backbone. Moreover, the sequence-dependent fine structure of the double helix is subtle and especially challenging to reproduce. For example, only in recent years, following improved representations of the sugar ring and its chemical links to the base and phosphate (Zgarbová et al. 2015; Ivani et al. 2016), has popular simulation software been able to bring the predicted twist-angles of individual base-pair steps into reasonable agreement with the average values of twist collected from well-resolved protein-DNA complexes (Todolli et al. 2017).

The need for reliable benchmarks increases as the technical barriers to atomic-level simulations of DNA are surmounted and studies of larger systems over longer and longer time periods become feasible. The credibility of the computed structures depends upon the extent to which the predictions match critical data. This article presents new standards gathered from the growing database of high-resolution X-ray crystallographic and cryogenic electron microscopy structures of protein-bound DNA. There is enough information now to characterize the configurational preferences of the DNA base-pair steps within the context of their immediate neighbors (Young et al. 2022) and to compare previously predicted effects of sequence context (Fujii et al. 2007; Pasi et al. 2014) with these findings. The crystallographic data also reveal a sequence-dependent buildup of different types of amino-acid atoms around the DNA bases (Olson et al. 2022) that potentially influences the observed configurations and recognition of DNA. State-of-the-art molecular simulations should aim to account for these properties as well.

### Early signs of sequence-dependent DNA structure

**Fig. 1:**
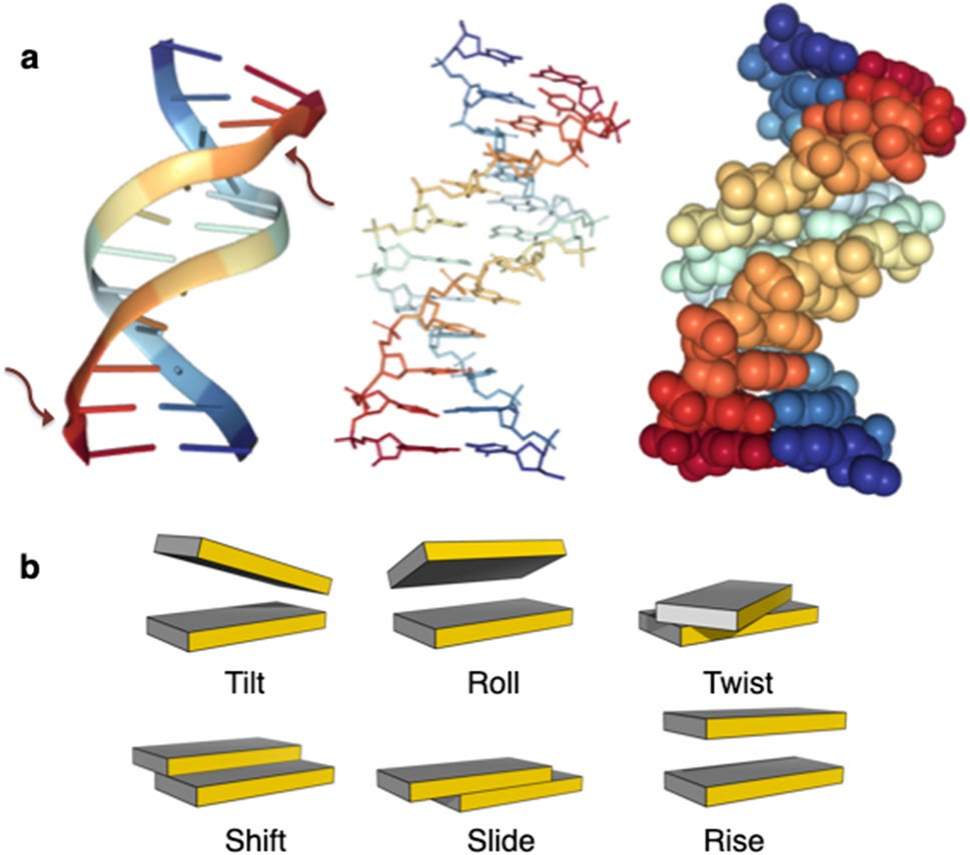
**a** Molecular representations of the 1.5-Å structure of the d(CGCGAATTCGCG)_2_ Dickerson-Drew DNA dodecamer (Sines et al. 2000). Images of Protein Data Bank file 1fq2 (Berman et al. 2000) rendered with the NGL viewer (Rose and Hildebrand 2015; Rose et al. 2018). GC dinucleotide steps found in the unusual BII form noted by arrows. **b** Pictorial representations of the six rigid-body parameters used to describe the orientation and displacement of successive base pairs. Images illustrate positive values of the designated parameters, with the sequence-bearing strand on the left and the minor-groove edge (gold) facing the reader

Although anticipated by the variety of helical models used to interpret the early X-ray fiber diffraction patterns of different synthetic polynucleotides (Chandrasekaran and Arnott 1989), the contributions of nucleotide sequence to DNA structure did not become clear until the determination of the first high-resolution crystal structure of the d(CGCGAATTCGCG)_2_ double helix, the so-called Dickerson-Drew dodecamer (Dickerson and Drew 1981). The gentle curve in the overall structure and the deformations of individual base pairs are visible to the eye in the derived atomic model (Fig. 1). Furthermore, unlike the regular models of fibrous DNA with identical arrangements of successive nucleotides, each base-pair step in the dodecamer adopts a different spatial state, both along the backbone and within the base pairs. Of particular note is the penultimate GC step along each of the self-complementary strands, which adopts a different helical form, termed BII DNA. The chain backbone undergoes large-scale conformational rearrangements at these steps along with changes in the twisting, bending, and lateral displacement of the attached base pairs.

The BII steps, noted by arrows in the illustration of one of the best-resolved dodecamer structures (Sines et al. 2000) (Fig. 1a), are overtwisted compared to the remaining steps of the molecule, and the base pairs do not align above one another in a perfectly parallel, overlapped fashion. The precise arrangement of the base pairs is described in terms of six rigid-body parameters (Fig. 1b)—three angles specifying the orientation of successive base-pair planes and the three components of the displacement vector joining successive base-pair centers (Dickerson et al. 1989; Olson et al. 2001; Lu and Olson 2003). The perturbed GC steps in the dodecamer stand out from the steps in the remainder of the molecule, with notably high values of Twist, negative values of Roll, and positive values of Slide—distortions attributed to intermolecular packing forces in the crystal lattice (Larsen et al. 1991). As noted below, the same type of large base-pair deformation occurs in the presence of proteins.

### Update on DNA sequence-dependent deformations

**Fig. 2:**
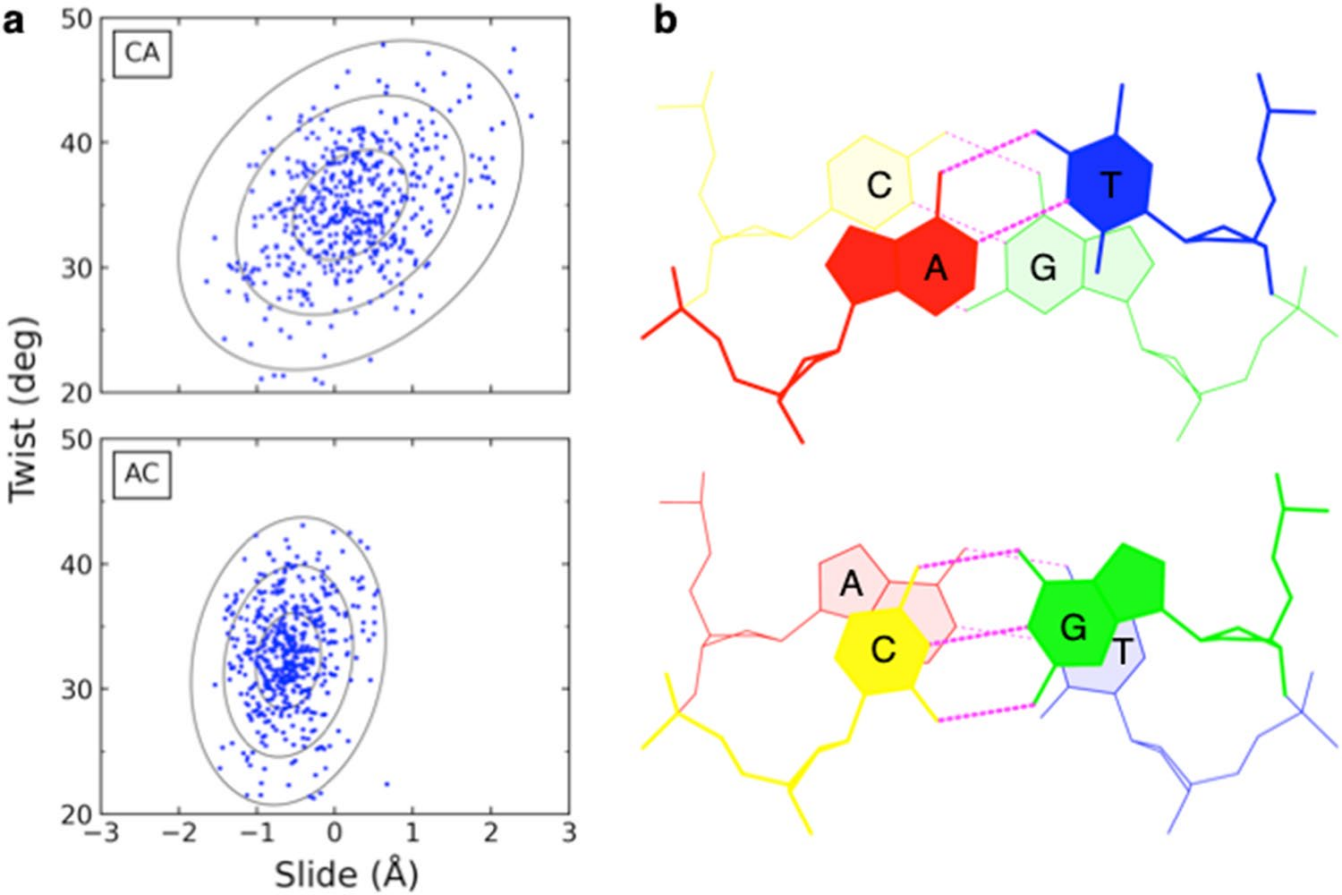
**a** Comparative scatter plots of the values of Slide and Twist found in a random sample of CA·TG and AC·GT base-pair steps taken from a recently curated set of non-redundant, high-resolution protein-DNA structures (Young et al. 2022). Ellipses are projections of the six rigid-body parameters on the Slide-Twist plane at 1σ, 2σ, and 3σ “energy” levels corresponding respectively to deviations of one, two, and three standard deviations from the average configuration. **b** Stacking diagrams, created with 3DNA (Li et al. 2019), illustrating the overlap and hydrogen-bonding patterns (dotted lines) in the corresponding base-pair steps. Base pairs fixed in ideal Watson–Crick arrangements (Olson et al. 2001), positioned in average configurations (Table 1), and linked by the optimized backbones generated with the software. Nucleotides color-coded by identity—A red, T blue, G green, C yellow—with the 5′-nucleotide on the leading strand and the 3′-nucleotide on the complementary strand in lighter hues. See text for additional details

The observed spread of DNA base-pair step parameters in high-resolution protein structures provides one of the best available estimates of the natural, sequence-dependent structure and deformability of the double-helical molecule (Olson et al. 1998).^1^ The rigid-body parameters of the ten unique base-pair steps cluster in distinctive, quasi-normal distributions consistent with harmonic behavior (see representative examples in Fig. 2a). The average values are suggestive of the rest state preferred by each dimer, and the pairwise covariance of variables—i.e., the differences between the mean squares and the squares of the means of all pairs of step parameters—shows how the configurations spread over the six parameters. This statistical perspective yields a smooth landscape that connects the many distinct structural examples and offers information about the likely overall motions of neighboring base pairs.

The average sequence-dependent twisting of base-pair steps found in a large, recently curated dataset of non-redundant protein-DNA structures (Young et al. 2022) follows the same order uncovered in early collections of DNA crystal structures (Gorin et al. 1995), with the twist of pyrimidine-purine, purine-purine, and purine-pyrimidine steps respectively increasing in the order CG < CA < TA, AG < GG < AA < GA, and AT < AC < GC (Table 1). These trends, which also occur in gels and in solution (Peck and Wang 1981; Kabsch et al. 1982; Tullius and Dombroski 1985), reflect clashes between the exocyclic atoms on the exposed edges of adjacent base pairs in combination with conformational restrictions on the sugar-phosphate backbone, namely the tendency of DNA to conserve the C1′ ⋅⋅⋅C1′ distances between the points of attachment of successive sugars to the base side groups (Calladine 1982; Gorin et al. 1995). As noted above for the Dickerson dodecamer, the twisting of base pairs is strongly correlated with Slide and Roll. One of the sources of these correlations lies in the apparent rigidity of the sugar-phosphate backbone. Changes in Twist, which directly alter the C1′⋅⋅⋅C1′ distance, must be tied to other base-pair step parameters to preserve the backbone constraints. An increase of Twist accordingly leads to a decrease in Roll and an increase in Slide. Thus, the values of Twist imply the values of other rigid-body parameters, as exemplified by the mean rigid-body parameters of the DNA base-pair steps in ~ 3900 X-ray crystallographic and cryogenic electron microscopic protein-DNA structures of 3.0 Å or better resolution (Table 1).

**Table 1:** Rigid-body parameters characterizing the average spatial arrangements of the ten unique DNA base-pair steps in high-resolution protein-DNA structures^†^

	Counts	Tilt (deg)	Roll (deg)	Twist (deg)	Shift (Å)	Slide (Å)	Rise (Å)
CG	5432	0	6.4	34.3	0	0.36	3.34
CA	3830	0.0	5.6	35.1	− 0.06	0.18	3.33
TA	4904	0	2.3	37.5	0	0.20	3.35
AG	3634	− 0.4	3.6	32.2	− 0.03	− 0.34	3.30
GG	3743	0.0	4.9	33.2	− 0.01	− 0.33	3.36
AA	3967	0.0	− 0.2	35.1	0.02	− 0.27	3.24
GA	4114	− 0.1	1.9	36.3	− 0.02	− 0.11	3.28
AT	5830	0	0.1	30.7	0	− 0.69	3.22
AC	3634	0.2	1.7	31.9	0.00	− 0.64	3.26
GC	4918	0	2.7	33.3	0	− 0.45	3.29

^†^Data based on a recently curated set of ~ 3900 non-redundant, high-resolution protein-DNA structures of 3 Å or better resolution (Young et al. 2022). Counts refer to the number of structural examples of the listed steps. Steps described in terms of the nucleotides on the leading strand

The correlated variations in base-pair step parameters become even clearer in scatter plots of representative data, here the values of Twist and Slide for 512 CA and 512 AC steps randomly selected from the aforementioned set of structures (Fig. 2a). The plotted data include 32 examples of each dimer in all 16 unique tetramer settings, i.e., with each dimer surrounded by all possible neighboring base pairs. The subset of data, corresponding to ~15% of the collected CA and AC steps, scatter in relatively random distributions around the mean values of Twist and Slide determined for the complete dataset (Table 1). Note the negative values of Slide that accompany the lower values of Twist in AC vs. CA steps. The most likely deformations of structure lie along the longest principal axes of the plotted data, i.e., along the long axes of the depicted ellipses. The contours of the ellipses correspond to 1*σ*, 2*σ*, and 3*σ* states deformed respectively by one, two, and three standard deviations from the average Twist and Slide values. The slopes of the axes reflect the sign of the correlation, e.g., the positive correlation of Twist with Slide in CA and AC steps. The magnitude of the slope reflects the strength of the correlations, e.g., more pronounced coupling of parameters for CA than AC steps. The natural deformations are a composite of all six rigid-body parameters based on the directions of the principal axes of the observed data. For example, each 1-Å increase in Slide along the largest principal axis of the depicted CA steps leads to a 0.2° decrease in Tilt, a 14.8° decrease in Roll, a 12.0° increase in Twist, a 0.11-Å decrease in Shift, and a 0.05-Å increase in Rise.

The average structures, constructed from the mean parameters of each base-pair step (Fig. 2b), highlight the differences in base overlap and twist associated with the reversal of pyrimidines and purines in dinucleotide sequences. The total overlap areas of the depicted base pairs increase from 2.7 to 10.8 Å^2^ with the switch from CA to AC steps. The leading CA- and AC-bearing strands, on the left side of each image, point out of the plane of the page and the complementary TG- and GT-bearing strands, on the right side, into the plane. The greater twist of the CA steps over the AC steps is evident from the greater angle between the sets of dotted lines depicting the hydrogen bonds on successive base pairs. The wider range of CA vs. AC deformations follows from the lesser overlap, i.e., weaker stacking interactions (Ornstein and Fresco 1983) of the pyrimidine-purine compared to the purine-pyrimidine dimer. Many of the large positive values of CA Twist and Slide depicted in Fig. 2b occur in concert with BII DNA backbone states, such as the kink-and-slide deformations found in the nucleosome core particle structure (Olson and Zhurkin 2011). Whereas the CA step appears to fluctuate with fairly comparable ease between under- and overtwisted states, the AC step rarely adopts overtwisted states (Fig. 2a).

### Effects of sequence context on DNA structure and deformability

The large number of high-resolution protein-DNA structures in the updated dataset makes it possible to characterize the configurational preferences of DNA base-pair steps within the context of their immediate neighbors (Young et al. 2022). The data include 130–472 examples of CA steps, depending on tetrameric setting, and 148–382 such examples of AC steps. The flanking base pairs have relatively limited effects on the average configuration of the central dimers. For example, the CA step is overtwisted on average relative to the 10.6-bp repeat of mixed-sequence DNA in most settings, the exceptions being the average twist values of CA in TCAA and TCAT tetramers, which are only slightly (< 1°) lower than that of a duplex with a 10.6-bp repeat (Table 2). The AC steps, by contrast, are consistently undertwisted relative to mixed-sequence DNA. Interestingly, the AC dimers within AACT sequences are among the least undertwisted AC steps, with an average value of Twist comparable to that of the CA steps in the reversed TCAA sequence. The average twist of CA in other CA-bearing sequences exceeds that of the AC in the sequence-reversed counterpart, i.e., xCAy vs. yACx, where ﻿x and y﻿ = A, G, C, T.

**Table 2:** Twist, in degrees, of CA·TG and AC·GT base-pair steps in all tetrameric contexts within high-resolution protein-DNA structures^†^

	CA steps					AC steps			
	AA	AG	AC	AT		CA	CG	CC	CT
AC	34.6	34.7	35.5	36.5	AA	32.6	33.4	33.6	33.3
GC	35.5	34.7	34.3	37.3	GA	31.4	31.0	32.1	32.1
CC	35.3	35.9	34.7	36.4	CA	30.5	31.1	31.2	31.7
TC	33.3	35.0	34.8	33.2	TA	30.7	32.4	31.3	32.1
35.1 ± 1.1					31.9 ± 1.0				

^†^Data from (Young et al. 2022). Average dimeric values (bottom line), based on equal weighting of each step in all tetrameric contexts

**Fig. 3:**
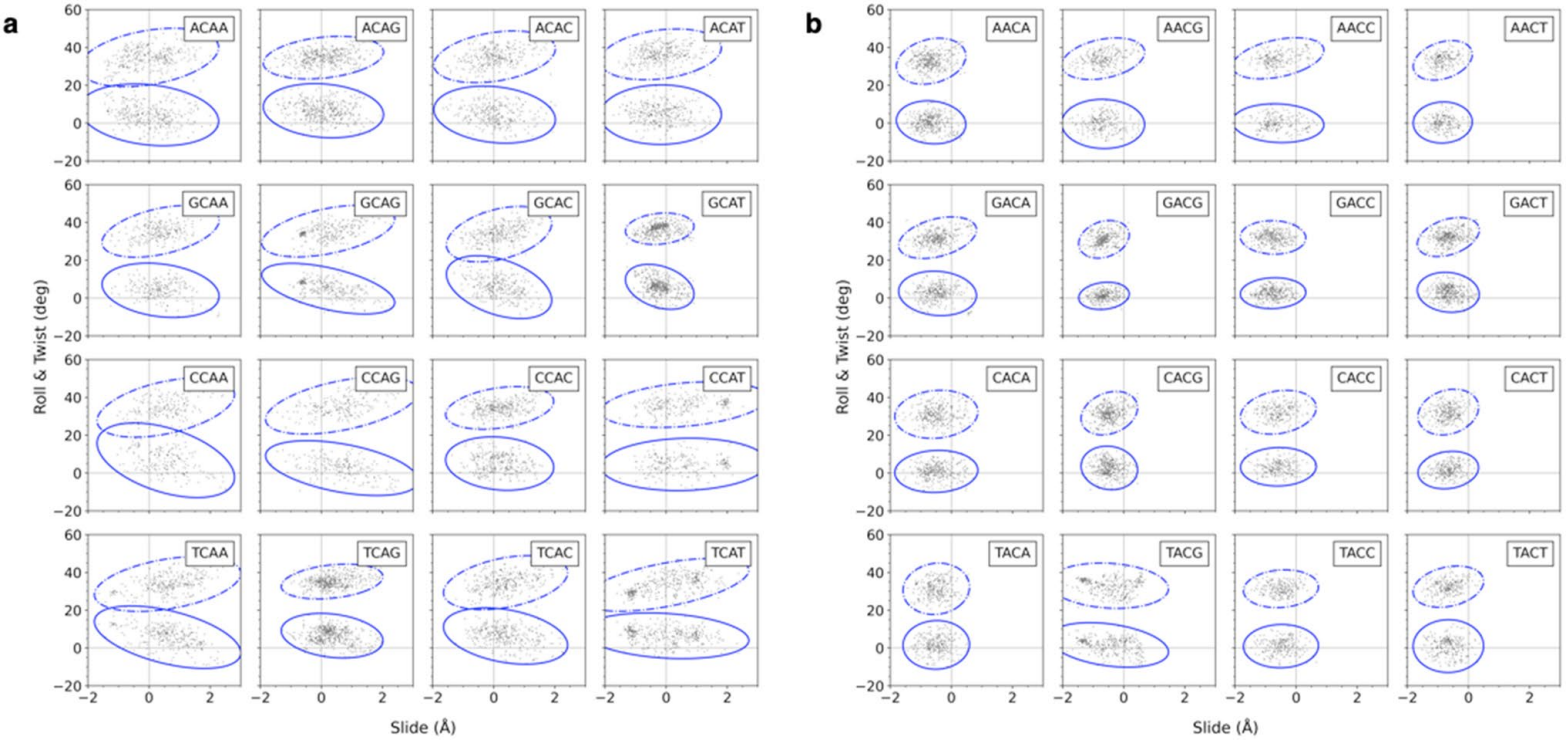
Scatter-plot arrays of the Slide, Roll, and Twist values adopted by **a** CA·TG and **b** AC·GT base-pair steps in all tetrameric contexts. Observed values of Roll and Twist respectively enclosed within solid and dashed contours at the 3σ level. Values observed in each tetrameric context placed in a higher-order 4 × 4 array such that the grids along each row share a common 5′-neighbor and those in each column a common 3′-neighbor. Data taken from (Young et al. 2022)

The characteristic deformability of different base-pair steps also persists in most tetrameric settings, with the range of CA deformations in Twist-Roll-Slide space fairly similar in all contexts save for those within GCAT tetramers (Fig. 3a). The area of Twist-Slide space spanned by the CA steps in the latter setting is less than half that spanned by the CA steps in most other sequence contexts. The range of occupied Roll-Slide space similarly drops in GCAT tetramers compared to the ranges found within other sequences. By contrast, the typically stiff AC step becomes more flexible within TACG sequences (Fig. 3b)—the reverse of the GCAT sequence that stiffens CA steps. These differences in deformability give rise to the variation in the areas of the 3*σ*-ellipses enclosing the values of Slide, Twist, and Roll adopted by CA and AC dimers in each tetrameric setting, e.g., the much smaller areas occupied by CA dimers within GCAT tetramers compared to those of CA dimers in other settings and the relatively larger area filled by AC dimers in TACG settings. Here, the values of Twist and Roll adopted by the central dimer in each tetrameric sequence are plotted vs. Slide, enclosed within a pair of 3*σ*-ellipses on the individual grids, and placed in a higher-order 4 × 4 array such that the grids along each row share a common 5′-neighbor and those in each column a common 3′-neighbor.

Some of the data clusters in small distinct regions significantly displaced from the elliptical centroids, observations suggestive of highly stable states or even two-state behavior. Note, for example, the apparent local preference of CA steps to adopt undertwisted states in GCAG settings and overtwisted states in CCAT settings and the apparent bimodality of CA steps in TCAT settings. The latter step appears to jump between under- and overtwisted states near 30° and 40° Twist with concomitant changes in Slide and Roll.

The set of images also reveals changes in rigid-body coupling that accompany changes in dimer deformability. The correlations of Twist, Roll, and Slide, for example, become weaker as the CA steps stiffen within a GCAT sequence and stronger as the steps become more flexible in other settings. Note the reorientation of the 3*σ*-ellipses, where the slope of the longest principal axis approaches zero as the ellipses decrease in area and becomes more positive or negative as the ellipses broaden. The degree of coupling is less regular for AC steps. The differences in average rigid-body parameters among the different tetramer settings give rise to the slightly shifted positions of the ellipses on the different scatter plots.

### Sequence-dependent DNA “solvation”

The examination of high-resolution protein-DNA structures further suggests that the nucleotides may contribute to the distribution of solvent molecules in the surrounding microenvironment. From the perspective of DNA, a bound protein is simply an entity presenting polar atoms capable of forming hydrogen bonds, charged atoms resembling free cations and anions, and nonpolar atoms with hydrophobic properties. When considered in such terms, a DNA-bound protein reduces to a highly ordered solvent molecule containing different types of relatively rigidly placed atomic species. In other words, the protein acts as a solvent cage fixed in place through association with DNA and limited in movement through its polypeptide chemical architecture.

**Fig. 4:**
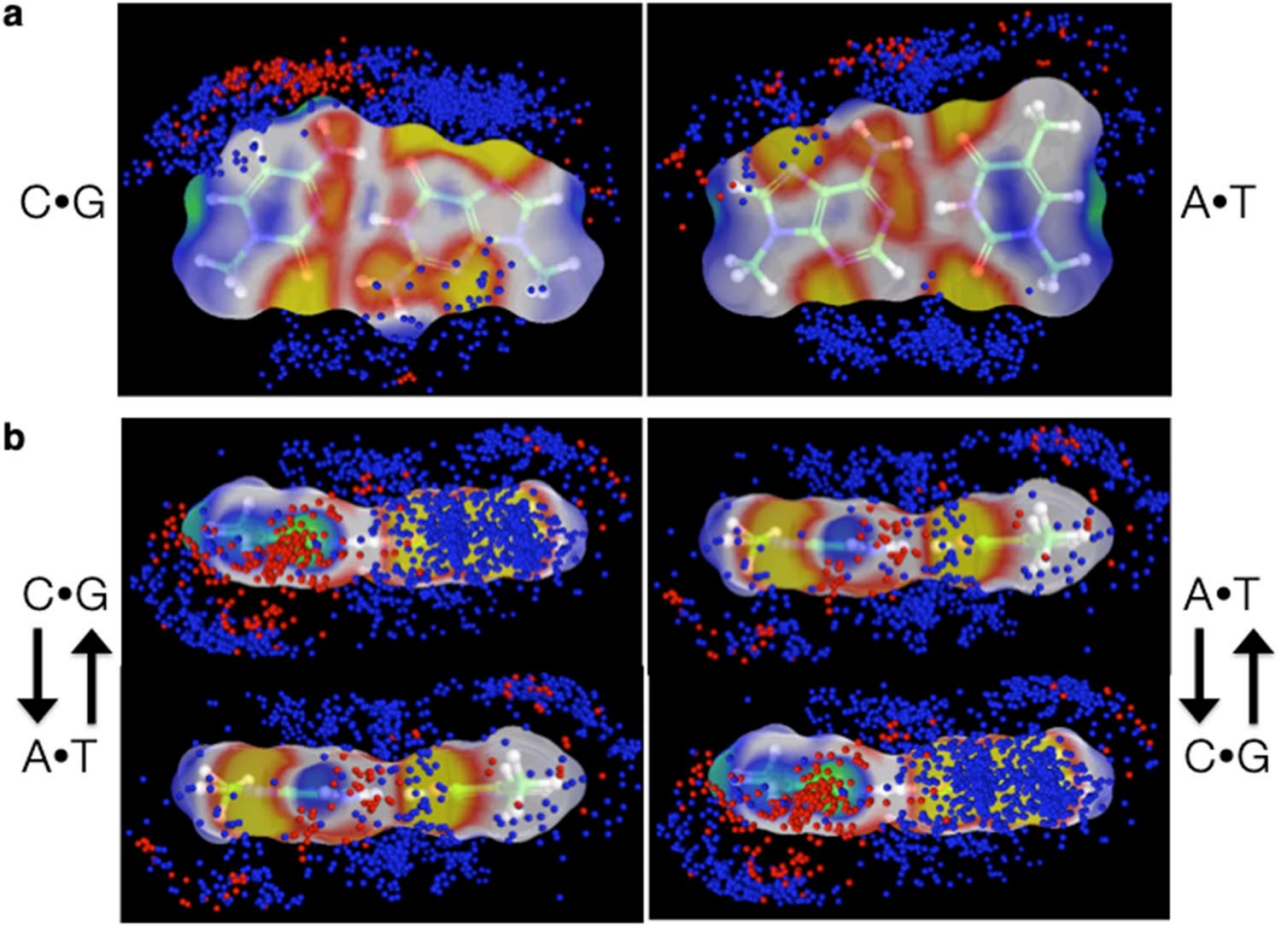
**a** Local ionic environment around C·G (left) and A·T (right) base pairs in high-resolution protein-DNA complexes superimposed on electrostatic potential surfaces of the base pairs (Srinivasan et al. 2009). Protein atoms within 4 Å of atoms in the base-pair fragments colored-coded by chemical type: cationic (blue) and anionic (red). View looking perpendicular to the base-pair plane with major- and minor-groove atoms located respectively on the top and bottom. White wireframe models of base pairs superimposed on solvent-excluded potential surfaces, with areas of greatest negative potential in yellow, greatest positive potential in green, and intermediate regions in red, white, and blue, respectively. **b** Major-groove views of the buildup of charged amino-acid atoms between the planes of successive C·G and A·T base pairs. Sugar-phosphate linkages of untwisted CA·TG (left) and AC·GT (right) steps depicted by arrows running from 5′- to 3′-nucleotides. Images taken from the video provided by (Olson et al. 2022)

Although the microenvironment presented by each protein around DNA is highly unique, the placement of different types of amino-acid atoms within close range of DNA in a small collection of non-redundant, high-resolution protein-DNA structures shows clear sequence dependence (Olson et al. 2022). The different chemical environments mirror the electronic properties of the base pairs, which, although electronically neutral, show a distinct buildup of charge around electronegative atoms and a reduction of charge around electropositive atoms (Srinivasan et al. 2009). The different placement of atoms on the four bases generates sequence-dependent electrostatic signatures that seemingly guide the accumulation of surrounding atomic species. For example, cationic atoms, such as the ammonium nitrogen on lysine, cluster in the vicinity of more electronegative sites and anionic atoms, such as the carboxylic oxygen of glutamic or aspartic acid, near more electropositive sites (Fig. 4a). Moreover, the greatest buildup of cations (blue dots) occurs in the vicinity of recognition sites with greatest negative (yellow) electrostatic potential and the greatest buildup of anions (red dots) in regions of greatest positive (green) electrostatic potential, most notably along the major-groove edges of G⋅C pairs near the electronegative O6 and N7 atoms of guanine and the electropositive N4 and C5 atoms of cytosine.

While most of the amino-acid atoms in contact with the bases lie on the outer edges of the base pairs, a sizable fraction occupy “intercalation” sites between the planes of successive base pairs. A large number of these examples involve cationic atoms in the vicinity of pyrimidine C5 atoms above the upper, or so-called primary (Lavery et al. 1992), faces of cytosine and thymine, and immediately below the next nucleotide. A number of out-of-plane contacts also occur above the upper faces of purines, albeit closer on average to the base-pair plane. The different accumulation of binding sites leads, in turn, to differences in the buildup of amino-acid atoms between the planes of pyrimidine-purine vs. purine-pyrimidine base-pair steps (Fig. 4b), here illustrated by the stacking of the ion clouds found within 4 Å of individual C⋅G and A⋅T base pairs. The base pairs are shown edge-on, with the atoms in the major groove facing outward toward the reader and the sugar-phosphate linkages depicted by arrows running from the 5′- to 3′-nucleotides on each strand. The contacts on the upper faces of C and T fill the middle of a CA⋅TG step but lie on the exterior of an AC⋅GT step (Fig. 4b, left vs. right). The sites of atomic uptake within the CA and TG steps may help to fill some of the empty space between the non-overlapped C⋅G and A⋅T base pairs.

These findings, based on 233 protein-DNA structures of 2.5 Å or better resolution, are consistent with observations found in larger structural datasets (Yu et al. 2019; Biedermannová et al. 2022). Understanding how the contact patterns might be tied to sequence-dependent structural features of DNA or to different types of proteins—e.g., the extent to which solvation patterns might contribute to base-pair overlap and deformability—requires additional information, both additional structural examples and insights from reliable molecular simulations.

### Prediction vs. observation

The physics-based force fields used in atomic-level simulations of DNA have the potential to uncover the forces that give rise to the sequence-dependent structural features found in high-resolution structures, such as why the CA base-pair step appears to stiffen in the context of a GCAT tetramer or seemingly to jump between under- and overtwisted forms within TCAT sequences. Simulations of small DNA duplexes initiated over 20 years ago pointed out the potential influence of sequence context on the local structure and deformability of DNA (Beveridge et al. 2004; Dixit et al. 2005). The simulations of collective atomic movements—performed over increasingly longer time periods within a surrounding cloud of water, potassium cations, and chloride anions (Fujii et al. 2007; Lavery et al. 2010; Pasi et al. 2014)—have reached the point where one can investigate the features of individual base-pair steps within a hexameric context (Balaceanu et al. 2019), i.e., examine the contributions of flanking dimers on the properties of a central base-pair step. Data are routinely collected over microsecond time periods, generating enormous numbers of configurational snapshots and sampling states outside the bounds typically imposed in analyses of high-resolution data, such as highly deformed base pairs and base-pair steps (Olson et al. 1998; Young et al. 2022).

Given that most of the aforementioned simulations were performed prior to the force-field improvements that take reasonable account of the observed sequence-dependent variation in dinucleotide Twist (Zgarbová et al. 2015; Ivani et al. 2016), many of the predictions do not match the observations reported herein. The systematically predicted undertwisting of pyrimidine-purine steps compared to experiment along with differences in simulation conditions may contribute to some of the predicted differences in the relative deformability of base-pair steps in different tetrameric contexts. For example, the CA base-pair steps generated in a series of 10-ns simulations of dodecamers are stiffened in the context of CCAA and CCAG tetramers (Fujii et al. 2007) whereas those produced within 18-mers over 100–1000-ns simulations are stiffened in the context of ACAT and ACAA tetramers (Lavery et al. 2010; Pasi et al. 2014). Neither the former prediction, based on the volumes of configuration space occupied by the simulated dimer steps, nor the latter prediction, estimated from the reported variances in Twist and Slide values, match the experimentally observed stiffening of CA steps in the context of GCAT tetramers. On the other hand, a number of the simulations draw attention to the bimodality of pyrimidine-purine base-pair steps, particularly the conformational interchange between canonical (BI) DNA and the BII form. The predicted examples include CA steps flanked by two pyrimidines (Lavery et al. 2010; Pasi et al. 2014) of the type suggested by the bimodal distributions of Twist, Roll, and Slide in TCAT sequences. The predicted frequency of occurrence of BII forms of CA steps, however, is much lower than that of AC steps, contrary to the observed distributions of base-pair step parameters in high-resolution structures.

The collection of experimental structures does not include the large proportion of undertwisted base-pair steps found to occur within particular nucleotide sequence contexts in simulations based on recent-generation DNA force fields (Zgarbová et al. 2017; Dans et al. 2019; Walther et al. 2020; Liebl and Zacharias 2021; Dohnalová and Lankaš 2022). Low-twisted states tend to occur in combination with base-pair “melting” in the observed protein-bound DNA structures. That is, one or more of the rigid-body parameters describing the orientation and displacement of complementary bases at untwisted base-pair steps—so-called buckle, propeller twist, opening, shear, slide, and stagger (Dickerson et al. 1989) — tend to lie three or more standard deviations from the mean values found for all base pairs. The violations of these limits exclude the vast majority of experimentally observed examples of DNA untwisting and thereby remove low-twist “bumps” of the type seen in the distributions of twist reported in many simulated structures.

The fact that the distributions of complementary base-pair parameters in the simulated structures span ranges of the same or lesser magnitude than those found in the culled experimental dataset (Dans et al. 2019; Dohnalová and Lankaš 2022) suggests that the untwisted steps in the simulated structures are not melted. The very low level of hydrogen-bond loss, or fraying, of Watson–Crick base pairs in these structures (Dans et al. 2019) supports this premise. The forces used to enforce hydrogen bonding, i.e., the choice of partial atomic charges, may be too strong in the simulated DNA structures. The set of partial atomic charges on the DNA bases is typically compared against quantum mechanical models of base separation along the long axis of an ideal Watson–Crick base pair, e.g., (Liebl and Zacharias 2023), as opposed to the range of base-pair deformations observed experimentally. In addition, there are C-H⋅⋅⋅O bonds that stabilize simulated, overtwisted BII states (Balaceanu et al. 2017), which, if too strong, would place too much weight on high-twist states. On the other hand, some have argued recently that hydrogen-bonding forces may not be strong enough in simulations of RNA (Kührová et al. 2019). The role, if any, of bound proteins in the “melting” of DNA base-pair steps found experimentally remains to be determined.

It should be noted that the algorithm used to obtain base-pair step parameters in the current work differs from that used to describe DNA in a number of simulations (Beveridge et al. 2004; Dixit et al. 2005; Lavery et al. 2010; Pasi et al. 2014; Dans et al. 2019; Walther et al. 2020; Liebl and Zacharias 2021). The differences lie in the approaches used to describe the rotations of base-pair reference frames. The data reported herein follow the engineering/physics-based perspective (El Hassan and Calladine 1995) implemented in the 3DNA software (Lu and Olson 2003). The description of base-pair geometry in many simulated structures follows the mathematical perspective implemented in the Curves + software (Lavery et al. 2009). Rotations are described with abstract coordinates involved in the Euler-Rodrigues formula, that obey the symmetry relations of the Cambridge convention (Dickerson et al. 1989) and have qualitative correspondence to conventional angles (Lankas et al. 2009). The values of Twist determined with Curves + differ from the values reported herein, with the differences increasing in magnitude as the DNA undergoes large deformations—e.g., differences in Twist of ~ 1° in steps resembling canonical (10.4–10.7 bp/turn) helical structures and 2–3° in overtwisted, kink-and-slide states of the type found in the nucleosome core particle (Olson and Zhurkin 2011).

Many DNA simulations have focused on the movement and accumulation of solvent molecules within and around the double-helical structure, with longer, more recent studies making it possible to examine the buildup of cations around specific oligonucleotide sequences and in different sequence contexts (Lavery et al. 2014; Pasi et al. 2015; Savelyev and MacKerell Jr 2015; Kolesnikov et al. 2021). The simulations capture the same patterns of cationic positioning seen in high-resolution structures, i.e., near the major-groove atoms of guanine and the minor-groove atoms of adenine, but have yet to keep track of the anions found to cluster around the amino groups of cytosine and adenine in high-resolution structures. Like the predictions of sequence-dependent structure and deformability, predictions of groove occupancy depend upon the simulation scheme—e.g., solvent model, DNA force field, and simulation time—along with the criteria used to define the presence of water, cations, and anions.

The observed patterns of protein-atom buildup in high-resolution structures provide useful new benchmarks for detailed atomic-level simulations of DNA, such as the relative populations of polar vs. charged atoms, i.e., water vs. cations, near different parts of DNA and the sites of preferential solvent accumulation. Recent progress in DNA force field development has taken advantage of the experimentally observed sequence-dependent double-helical structure in the validation of simulations (Zgarbová et al. 2015; Ivani et al. 2016), but not the apparent organization of different solvent species around DNA. The sites of atomic buildup, described in standard coordinate frames on the bases, can be compared with the locations of solvent atoms in similar frames on simulated structures. Future simulations that take account of both the known sequence-dependent structural features of DNA and the distribution of solvent molecules in the local microenvironment hold promise for deciphering the forces that stabilize and destabilize the double-helical molecule as it folds and functions in biological settings.

## Data Availability

Not applicable. This article is a review article.

## Footnotes

1 There are not sufficient data to address the sequence-dependent deformability of ligand-free DNA. Crystallographic structures are limited to a relatively small number of sequences and few space groups, and there are no well-resolved cryo-electron microscopic examples to date.
